# Identifying the culprit lesion in tumor induced hypophosphatemia, the solution of a clinical enigma

**DOI:** 10.1007/s12020-016-1092-5

**Published:** 2016-10-05

**Authors:** Mathilde M. Bruins Slot-Steenks, Neveen A.T. Hamdy, Michiel A.J. van de Sande, Dennis Vriens, Arjen H.G. Cleven, Natasha M. Appelman-Dijkstra

**Affiliations:** 1 Center for Bone Quality and Department of Medicine, Division of Endocrinology, Leiden University Medical Center, Albinusdreef 2, Leiden, 2333 ZA The Netherlands; 2 Center for Bone Quality and Department of Orthopedic Surgery, Leiden University Medical Center, Albinusdreef 2, Leiden, 2333 ZA The Netherlands; 3 Department of Radiology, Leiden University Medical Center, Albinusdreef 2, Leiden, 2333 ZA The Netherlands; 4 Department of Pathology, Leiden University Medical Center, Albinusdreef 2, Leiden, 2333 ZA The Netherlands

**Keywords:** Tumor-induced osteomalacia (TIO), Fibroblast growth factor 23 (FGF23), Hypophosphatemia

## Abstract

Tumor-induced osteomalacia is a rare acquired metabolic bone disorder characterized by isolated renal phosphate wasting due to abnormal tumor production of fibroblast growth factor 23. We report the case of a 59 year old woman referred to our department with a long history of progressive diffuse muscle weakness and pain, generalized bone pains and multiple insufficiency fractures of heels, ankles and hips due to a hypophosphatemic osteomalacia. A fibroblast growth factor 23-producing phosphaturic mesenchymal tumor localized in the left quadriceps femoris muscle was identified 7 years after onset of symptoms. Excision of the tumor resulted in normalization of serum phosphate and fibroblast growth factor 23 levels and in complete resolution of the clinical picture with disappearance of all musculoskeletal symptoms. This case illustrates the diagnostic difficulties in establishing a diagnosis tumor-induced osteomalacia and in identifying the responsible tumor. Our case underscores the clinical need to investigate all patients with persistent musculoskeletal symptoms for hypophosphatemia. A systematic approach is of pivotal importance because early recognition and treatment of the metabolic abnormality can prevent deleterious effects of osteomalacia on the skeleton.

## Introduction

Tumor-induced osteomalacia (TIO) is a rare acquired metabolic bone disorder occurring as a result of isolated renal phosphate wasting due to abnormal tumor production of fibroblast growth factor 23 (FGF23). Lack of awareness of the clinical manifestations of the disorder often leads to diagnostic delay, failure of diagnosis or misdiagnosis. Once a diagnosis TIO is suspected it is also often difficult to find the responsible FGF23-secreting tumor, since these tumors are often small, slow growing, benign tumors of mesenchymal origin, which can occur almost anywhere in bone or soft tissue [1–4]. Since the source of the FGF23 production is often very hard to find careful follow-up is necessary. Regular restaging should be performed until the culprit lesion is detected [3, 5]. In this article we do not only discuss the intense long diagnostic workup and follow-up of a patient with TIO but we also show that when patients are carefully monitored this can be performed without complications, e.g., tertiary hyperparathyroidism due to phosphate suppletion or new fractures.

### Case description

**Fig. 1:**
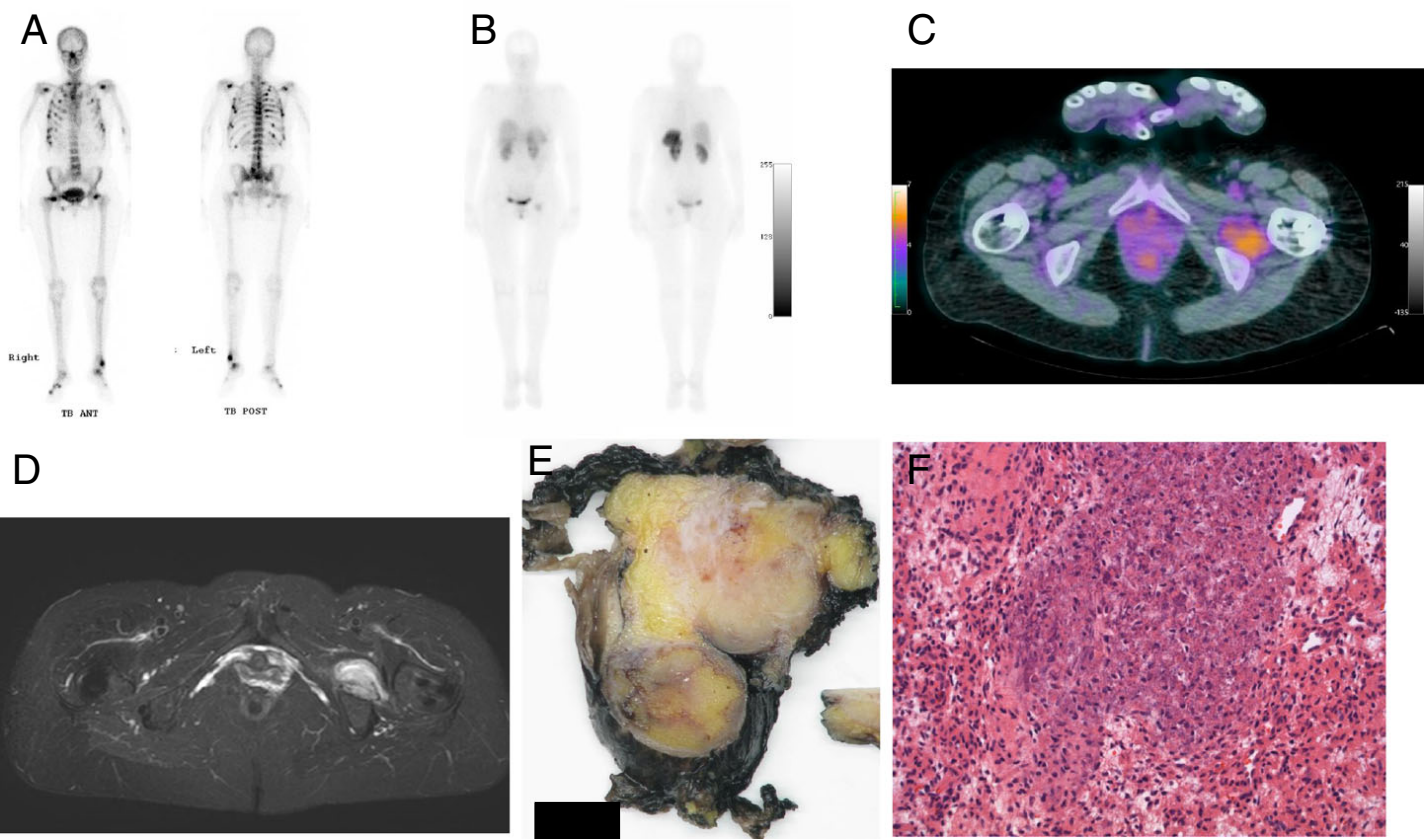
**a**. Skeletal scintigraphy performed 4 h after injection of ^99m^Tc-hydroxydiphosphonate showing multiple focal areas of increased osteoblastic activity suggestive for microfractures together with general increased osteoblastic activity throughout the whole skeleton, suggestive for a metabolic bone disease. **b**.^111^Indium-pentetreotide planar total body scintigraphy (24 h after injection) showing pathological somatostatin receptor expression in the left inguinal region. **c**. Fusion transversal image of the ^**18**^fluorodeoxyglucose positron emission tomography/computed tomography (FDG‐PET/CT) showing a soft tissue lesion with moderately increased glucose metabolism (maximum standardized uptake value (SUVmax) 4.2 g/cm^3^ 47 min post-injection of 175 MBq FDG) in the left inguinal region, situated in the m. quadrates femoris. On the full-body images, no other FDG-avid lesions were detected. **d**. Transversal short tau inversion recovery (STIR) magnetic resonance image with an intermuscular located soft tissue mass inferior to the left hip joint. The lesion demonstrates inhomogeneous high signal intensity compared to muscle. **e**. Macroscopy shows a well circumscribed nodular soft tissue tumor (diameter 6 cm). (black bar = 1 cm). **f**. Microscopy shows a tumor composed of bland, spindle to stellate cells, surrounding a matrix of phosphaturic mesenchymal tumor that typically calcifies in a type of ‘grungy’ or flocculent fashion

A 59-year-old woman with a longstanding history of epilepsy with complex partial seizures, for which she had been using phenytoin for more than 20 years, was referred to our center with a 3-year history of progressive muscle weakness, associated with generalized bone and muscle pain, resulting in significant limitations in her daily activities. The referring physician’s working diagnosis was osteoporosis secondary to long-term use of anti-convulsant therapy in the form of phenytoin. The diagnosis of osteoporosis was made on the basis of low bone mineral density measurements using dual-energy X-ray absorptiometry (lumbar *T*-score −2.2 standard deviations (SD), femoral neck *T*-score −3.0 SD) and stress fractures of heels and ankles. The patient was also found to have a vitamin D deficiency, low serum calcium and phosphate concentration (respectively 2.13 and 0.45 mmol/L) and elevated alkaline phosphatase (366 U/L). Treatment with phenytoin was discontinued. Supplementation of vitamin D in the form of colecalciferol (vitamin D3) and calcium carbonate was used in a dosage of 400 IE and subsequently 500 mg 3 times per day. Subsequently intravenous treatment with bisphosphonates (pamidronate) was initiated before referral to our center. In view of persistence of complaints, a ^99m^Tc-skeletal scintigraphy was performed, showing multifocal areas of increased radioactive isotope uptake, including at the femoral neck bilaterally, interpreted to be due to multiple insufficiency fractures (Fig. 1a). The patient was referred to our center for further investigation and management because persistence of symptoms and hypophosphatemia and inadequate response of bone mineral density to bisphosphonate after an year of therapy.

At presentation in our center, the patients symptoms had further worsened with progressive proximal muscle pain and weakness and increasing bone pain. She was still using calcium/vitamin D supplementation. Laboratory investigation confirmed the presence of severe hypophosphatemia. In retrospect this was also the case at first presentation elsewhere. She had a normal renal and liver function, the serum calcium was still low in the presence of a normal parathormone, normal 25-hydroxyvitamin D and a low normal 1,25-dihydroxy vitamin D (Table 1). Bone turnover was elevated as suggested by high alkaline phosphatase. Conventional radiographs demonstrated Looser’s zones in the femoral neck bilaterally. A diagnosis of hypophosphatemic osteomalacia was suspected. The 24-h urinary phosphate was 15 mmol/24 h. The fractional tubular reabsorption of phosphate (TRP) was low 0.77, calculated by the formula TRP = 1 − {(Urine phosphate × Serum creatinine)/(Serum phosphate × Urine creatinine)}. Second morning-void urine and blood samples are needed taken at the same time without phosphate supplementation. When TRP is ≤0.86 then the tubular maximum for phosphate corrected for glomerular filtration rate (TmP/GFR) can be calculated by the formula TmP/GFR = TRP × serum phosphate, which was remarkable low 0.5 mmol/L (normal range 0.88–1.42 mmol/L). TmP/GFR can also be determined using a nomogram [6]. The results were compatible with renal phosphate wasting. Considering the patient’s age, normal phenotype and negative family history for bone and mineral disorders, a diagnosis of tumor-induced osteomalacia was considered. The C-terminal FGF-23 (Immutopics, San Clemente, CA, USA) was measured in ethylene-diamine-tetraacetic acid (EDTA) plasma. The BioTek ELx50 (BioTek, Bad Friedrichshall, Germany) was used as an automatic washing machine. All analyses were performed according to manufacturer’s protocol [7]. Serum C-terminal FGF23 was found to be above the upper limit of the normal laboratory reference of 123 RU/mL (normal value <120 RU/ml), inappropriately high in the presence of hypophosphatemia. An extensive search using different imaging tools including ^111^Indium-pentetreotide whole body single photon emission tomography (SPECT), computed tomography (CT) and magnetic resonance imaging (MRI), revealed no potential lesion as a source of FGF23 production. The patient was treated with active vitamin D metabolite (alfacalcidol 1dd1.5 µg) and phosphate drink (3dd20 mmol), which partially increased serum phosphate level associated with improvement of symptoms. However, this was insufficient to control hypophosphatemia and associated symptoms in the long-term. There was a clear increase in C-terminal FGF23 (2050 RU/ml) over the following 4 years and it became progressively more difficult to control serum phosphate levels. Her clinical picture also significant worsened. She had difficulty in walking and surgical intervention was required to stabilize the severe insufficiency fractures of her hips. At that stage further radiological evaluation using ^111^Indium-pentetreotide scintigraphy and also a ^18^Fluorodeoxyglucose positron emission tomography/CT (FDG-PET/CT) was performed, both demonstrated a suspicious isolated lesion in the soft tissue at the left inguinal region (Fig. 1b, c). A MRI confirmed the presence of a small tumor in the left quadriceps femoris muscle (Fig. 1d), which was subsequently successfully resected. The resected lesion was a soft tissue tumor, with macroscopic features of a nodular tumor with a maximum diameter of 6 cm (Fig. 1e). Figure 1f shows the histology of the tumor composed of bland, spindle to stellate cells, surrounding a matrix of phosphaturic mesenchymal tumor that typically calcifies in a ‘grungy’ or flocculent fashion. Within 5 days after complete resection of the tumor, there was a rapid and complete normalization of serum phosphate and FGF23 levels associated with complete resolution of the clinical picture.

**Table 1:** Laboratory investigation

Laboratory findings	Initial results	At presentation in our center	3 years follow-up	4 years follow-up	Postoperative results (5 days after resection)	Reference range
phosphate (mmol/L)	0.45	0.56	0.53	0.65	1.18	2.15–2.55
calcium (mmol/L)	213	2.08	2.28	2.49	2.39	0.90–1.50
creatinine (µmol/L)	–	57	62	85	65	49–90
alkaline phosphatase (U/L)	366	271	115	148	102	0–98
PTH (pmol/L)	7.0	7.8	4.4	1.6	5.4	1.5–8.0
vitamin D 25(OH) (nmol/L)	nr	73	78	92	89	50–250
1,25 (OH) 2 vitamin D (pmol/L)	–	58	80	–	–	40–140
C-FGF23 (*RU/L)/(**U/L)	–	*123	**1370	**2050	**54	*0–120
						**0–125

*nr* not retainable

## Discussion

Tumor-induced osteomalacia was first described by McCance in 1947 [8]. In 1959 Prader et al. were the first to recognize that the disease was the result of a tumor which secreted a ‘rachitogenic’ substance and that resection of the tumor resulted in resolution of the osteomalacia [9]. A growing recognition of the disease paralleled the identification of FGF23 as a phosphaturic agent in 2000 [10]. Despite unraveling the pathophysiology of TIO, there is still often delay in establishing the diagnosis because associated musculoskeletal symptoms are non-specific, the FGF23-producing tumor is often very small and its localization very elusive. A delay in time between onset of symptoms and a further delay in localization and resection of the FGF23-producing tumor may be associated with considerable morbidity because of progressive osteomalacia if the hypophosphatemia remains untreated or is more difficult to control if treated with active vitamin D metabolites as the tumor growth increases production of FGF23, resulting in increasing renal phosphate wasting.

FGF23 is a hormone produced by osteocytes and osteoblasts. FGF23 acts primarily at the proximal renal tubule by inhibiting tubular phosphate reabsorption by reducing the expression of the sodium phosphate co-transporters NaPi-IIa and NaPi-IIc through activation of the receptor complex of fibroblast growth factor receptor 1c and its co-receptor klotho. FGF23 also inhibits the expression of the renal 1α-hydroxylase enzyme and decreases thereby 1,25-dihydroxy vitamin D production [11, 12]. The pathognomonic features of excess circulating FGF23 are renal tubular wasting of phosphate and suppressed 1,25-dihydroxy vitamin D levels which further impair intestinal absorption of phosphate worsening hypophosphatemia and resulting in impaired mineralization of bone.

A systematic approach to hypophosphatemia helps in the early recognition and treatment of TIO. Presenting symptoms of TIO are diffuse muscle and bone pain, initial proximal muscle weakness which becomes generalized and insufficiency fractures. A careful history taking and physical examination is important followed by laboratory investigations including calcium, phosphate, creatinine, parathyroid hormone (PTH) and vitamin D metabolites. All cases of hypophosphatemia should be further evaluated to establish whether low circulating levels of phosphate are due to renal phosphate wasting, decreased intestinal absorption of phosphate or to redistribution of phosphate from extracellular fluid into cells or into the bone matrix. Hypophosphatemia can also be caused by several medications. The underlying pathophysiological mechanism of medication-induced hypophosphatemia are as mentioned before or resulting from more than one mechanism. Our patient was known with prolonged therapy with anticonvulsants in the form of phenytoin. These drugs are inducers of the cytochrome P450 thereby causing increased vitamin D degradation. They also decrease calcium resorption in the gut [13]. In our case it was remarkable that the hypophosphatemia persisted after stopping phenytoin and vitamin D suppletion, so further evaluation was required. To determine if hypophosphatemia is the result of renal phosphate wasting it is necessary to calculate the TMP/GFR. Once renal phosphate wasting is determined the measurement of FGF23 is an important next step if parathyroid hormone concentration is in the normal range. FGF23 can be measured using two different assays, the intact FGF23 assay and the C-terminal FGF23 assay which detects intact FGF23 peptide and biologically inactive carboxyl terminal (C-terminal) fragments. Acquired hypophosphatemia due to renal phosphate wasting is most likely due to TIO, in which case excess FGF23 is most often produced by benign, small phosphaturic mesenchymal tumors [1–5, 14]. Malignant tumors and metastases are rare [5]. A benign phosphaturic mesenchymal tumor may occasionally progress to a high-grade osteosarcoma [15]. FGF23 secretion may also be observed in association with rare bone diseases such as neurofibromatosis, epidermal nevus syndrome and isolated fibrous dysplasia or in the setting of McCune-Albright [16–18]. Other possible causes of renal phosphate wasting with elevated FGF23 levels include inherited hypophosphatemic osteomalacia such as X-linked hypophosphatemic rickets, autosomal dominant hypophosphatemic rickets and autosomal recessive hypophosphatemic rickets. FGF23-independent causes of hypophoshataemia are alcohol abuse, drugs or toxins, renal tubular acidosis, Fanconi’s syndrome or hereditary hypophosphatemic rickets with hypercalciuria.

**Fig. 2:**
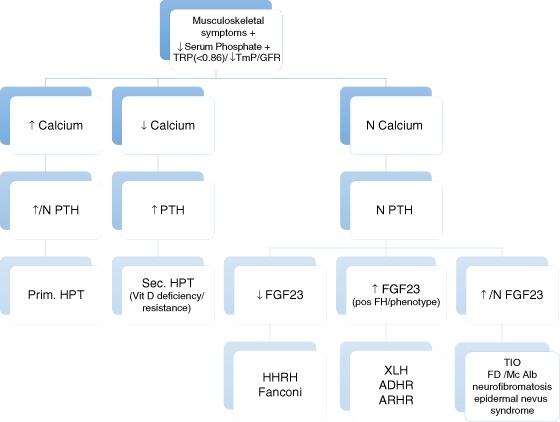
Suggested flowchart musculoskeletal symptoms and hypophosphatemia. *TRP* fractional tubular reabsorption of phosphate, *TmP/GFR* tubular maximum reabsorption of phosphate to glomerular filtration rate, *N* normal, *PTH* parathyroid hormone, Prim. *HPT* primary hyperparathyroidism, Sec. *HPT* secondary hyperparathyroidism, *FGF23* fibroblast growth factor 23, *FH* family history, *HHRH* hereditary hypophosphatemic rickets with hypercalciuria, *XLH* X-linked hypophosphatemic rickets, *ADHR* autosomal dominant hypophosphatemic rickets, *ARHR* autosomal recessive hypophosphatemic rickets, *TIO* tumor-induced osteomalacia, *FD* Fibrous dysplasia, *Mc Alb* McCune Albright syndrome

The finding of hypophosphatemia in the absence of a family history for bone and mineral disorders, a normal phenotype and relatively recent onset of symptoms which may rapidly progress in an adult with previously documented normal serum phosphate suggest TIO, which may be confirmed by measuring FGF23 levels (Fig. 2). The definitive diagnosis of TIO is established by identification of the causative tumor and by resolution of the clinical picture following complete tumor resection. Complete surgical resection of the FGF23 secreting tumor is the only definitive therapy, which emphasizes the need for localizing FGF23-producing tumors. Localization of FGF23-producing tumors can be challenging and a step-wise approach, including functional imaging, followed by anatomical imaging, and if necessary selective venous sampling may significantly improve success in tumor localization [4]. Tumors associated with osteomalacia variably express somatostatin receptors (SSTR1–5), allowing SSTR-based functional imaging by octreotide scintigraphy such as ^111^In-pentetreotide [19]. Because predominant overexpression of SSTR-2 subtype in tumors of TIO, ^68^Ga-octreotide PET/CT may have an important role in localization of the culprit lesion, and may potentially reduce significant delay in establishing the diagnosis of TIO [20]. FDG-PET/CT can confirm a lesion or identify a lesion that was not initially seen on octreotide scintigraphy [4, 20]. FDG-PET/CT has been proven to be sensitive but not a specific method of identifying FGF23-producing tumors. PET/CT using 68-gallium is expected to be much more specific and informative for this kind of tumors but is often not available in many centers [5]. Anatomic imaging is used to confirm the localization of a tumor. When multiple suspicious lesions are identified selective venous sampling can increase diagnostic power by confirming the localization of the culprit lesion [21]. Furthermore establishing the diagnosis of an FGF23-producing phosphaturic mesenchymal tumor requires an adequate integration of the clinical picture in correlation with radiology and histology in a multidisciplinary setting, since radiology and histology show considerable overlap with other entities that need different treatment and harbor other clinical outcomes.

Since identifying the source of FGF23 overproduction may be challenging and often inconclusive in patients with TIO, medical treatment should be initiated. Phosphate treatment alone will not be sufficient and can be counterproductive since oral phosphate stimulates PTH secretion and is poorly absorbed due to impaired 1,25-dihydroxy vitamin D hydroxylation. Furthermore, the compliance of oral phosphate is rather low since this is often not well tolerated. Therefore treatment with active vitamin D metabolites is necessary since FGF23 not only increases urinary phosphate loss but inhibits the expression of 1α-hydroxylase as well, leading to a decreased 25-hydroxyvitamin D to 1,25-dihydroxy vitamin D hydroxylation. Intestinal absorption of dietary phosphate will be impaired since the expression of type 2b sodium phosphate co-transporter is downregulated due to the lack of 1,25-dihydroxy vitamin D. Furthermore the decrease in 1,25-dihydroxy vitamin D stimulates the synthesis and secretion of PTH by the parathyroid glands thereby furthermore inducing urinary phosphate loss. By supplementation of active vitamin D metabolites you increase the intestinal absorption of phosphate and suppress PTH production and should therefore be the drug of choice. In cases the hypophosphatemia is severe oral phosphate can be added with close monitoring of the PTH levels and urinary calcium excretion since this will increase urinary calcium excretion which can lead to nephrocalcinosis and nephrolithiasis. Therapy should be continued for as long as the tumor is not identified or resected. Patients should also be closely monitored for complications of the hypophosphatemia, because longstanding hypophosphatemia can lead to progression of osteomalacia and associated increased risk of fractures. Adenosine triphosphate (ATP) depletion in hypophosphatemia can also effect the cardiopulmonary system by impaired myocardial and diaphragmatic contractility and effect the hematopoietic system leading to hemolysis and leukocyte dysfunction, these complications are rare [22]. The search for the source of FGF23 should also continue using state of the art imaging techniques.

Surgical intervention is curative when a lesion is identified and fully resected. Radical excision is important to avoid a recurrence. After successful tumor resection, the serum level of FGF23 decreases to a normal level within a few hours to days depending on the magnitude of the initial elevation (half-life of FGF23 is approximately 46–58 min) [23]. Improvement of clinical symptoms rapidly follows with subsequent complete remineralization of the skeleton [19]. Late recurrence due to local recurrence or distant metastasis is very uncommon and occurs in approximately less than 5 % of patients with TIO [2, 3]. Long-term follow-up is recommended, FGF23 can be used as a tumor marker for early detection of recurrences.

## Conclusion

Tumor-induced osteomalacia represent a significant diagnostic challenge and should be considered in the differential diagnosis of all patients with musculoskeletal symptoms associated with hypophosphatemia. Association of hypophosphatemia, renal phosphate wasting (low TRP/low TmP/GFR), elevated FGF23 levels and paradoxically low or normal 1,25-dihydroxy vitamin D suggests the diagnosis of TIO after exclusion of the hereditary hypophosphatemic osteomalacia by adult onset of hypophosphatemia in the absence of family history and characteristic phenotype. Awareness of this acquired metabolic abnormality, a systematic approach and evaluation of a hypophosphatemia in a multidisciplinary setting are of pivotal importance because early recognition and treatment can prevent progression of osteomalacia and associated increased risk of fractures. Patients should be prescribed active metabolites of vitamin D with a few requiring additional phosphate supplementation. After the FGF23-producing tumor is located and fully resected, long-term follow-up is advocated for early diagnosis of a potential recurrence.
